# Colonic Spirochetosis in a 60-Year-Old Immunocompetent Patient: Case Report and Review

**DOI:** 10.1177/2324709616662671

**Published:** 2016-08-12

**Authors:** Taiwo Ngwa, Jennifer L. Peng, Euna Choi, Sucharat Tayarachakul, Suthat Liangpunsakul

**Affiliations:** 1 Division of Gastroenterology and Hepatology, Department of Medicine, Indiana University School of Medicine, Indianapolis, IN, USA; 2 Department of Pathology and Laboratory Medicine, Indiana University School of Medicine, Indianapolis, IN, USA; 3 Roudebush Veterans Administration Medical Center, Indianapolis, IN, USA

**Keywords:** Spirochetosis, Colon, Immunocompetent host

## Abstract

Spirochetes, a genetically and morphologically distinct group of bacteria, are thin, spiral-shaped, and highly motile. They are known causes of several human diseases such as syphilis, Lyme disease, relapsing fever, and leptospirosis. We report a case of colonic spirochetosis in a healthy patient presenting for surveillance colonoscopy. The diagnosis of intestinal spirochetosis was made accidentally during the histological examination of colonic polyps, which were removed during colonoscopy. We also performed an extensive review on intestinal spirochetosis with a focus on clinical presentation and outcomes of reported cases from the past two decades.

## Introduction

Spirochetes are a genetically and morphologically distinct group of bacteria. Morphologically, they are thin, spiral-shaped, and highly motile.^1^ Spirochetes are known causes of several human diseases such as syphilis, Lyme disease, relapsing fever, and leptospirosis. Intestinal infestation by spirochetes has long been recognized.^2^ Clinical presentations vary, ranging from asymptomatic to gastrointestinal (GI)-related symptoms such as bleeding or diarrhea.^3^ We report a case of colonic spirochetosis in a healthy patient who initially presented for surveillance colonoscopy. Additionally, we also perform an extensive review of previously reported cases in the literature.

## Case Report

**Figure 1.**
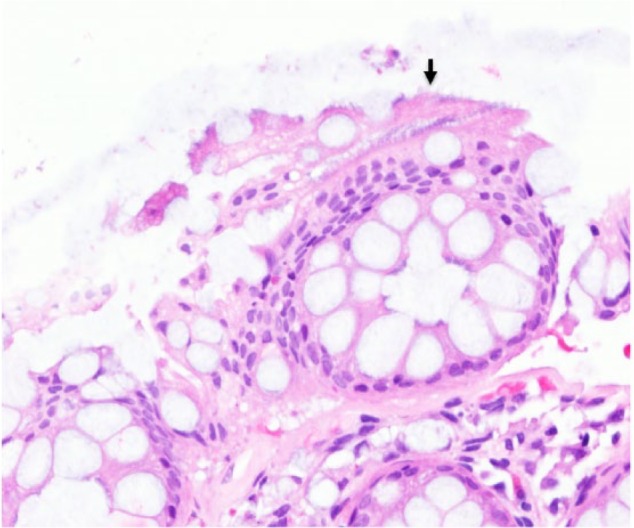
Intestinal spirochetosis on H&E stain (20×). Solid black arrow indicates spirochetes attached to the luminal side of colonic mucosa forming a “false brush border.”

**Figure 2.**
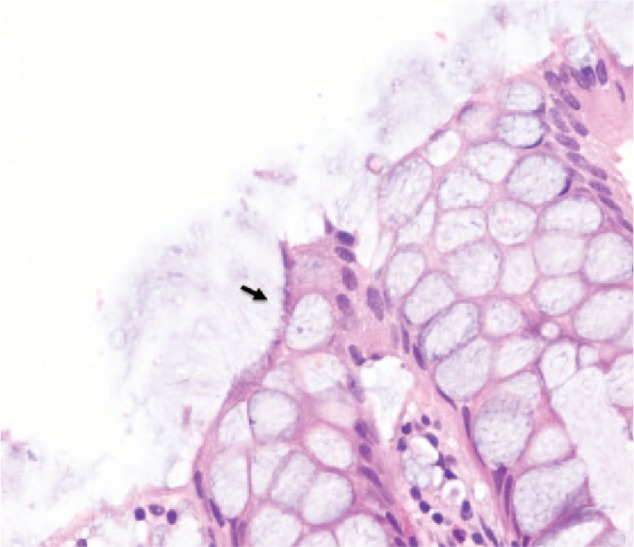
Intestinal spirochetosis on H&E stain (40×). Solid black arrow indicates spirochetes attached to the luminal side of colonic mucosa forming a “false brush border.”

**Figure 3.**
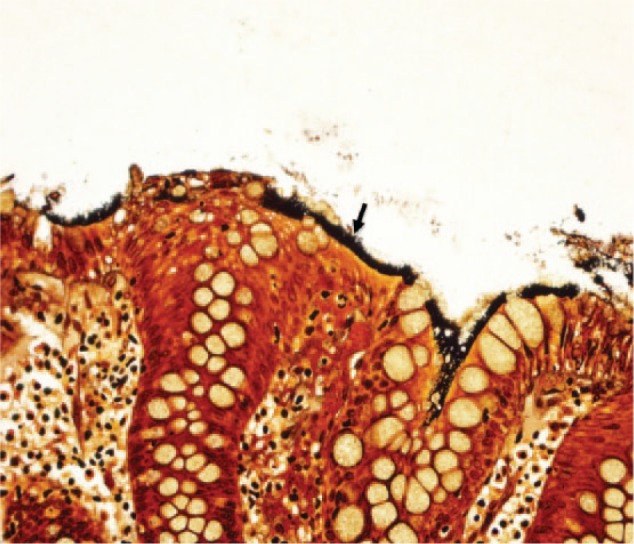
Intestinal spirochetosis on Warthin-Starry (silver) stain (40×). Solid black arrow indicates spirochetes attached to the luminal side of colonic mucosa forming a “false brush border.”

A 60-year-old asymptomatic man with no significant past medical history underwent a surveillance colonoscopy due to a previous history of a 1.8-cm hyperplastic polyp at the ileocecal valve. He denied weight loss and any GI symptoms, such as abdominal pain, diarrhea, or rectal bleeding. Colonoscopy revealed 2 tubular adenoma polyps in the cecum and 6 hyperplastic polyps in the rectosigmoid junction, ranging from 2 to 4 mm. The hematoxylin and eosin (H&E) stain of these polyps showed several filamentous structures on the colonic epithelium (Figures 1 and 2). A Warthin-Starry stain was subsequently performed and confirmed the diagnosis of intestinal spirochetosis (Figure 3). He also tested negative for HIV (human immunodeficiency virus) infection.

## Discussion

Intestinal spirochetosis (IS), first described by Harland and Lee in 1967 using electron microscopy,^4,5^ is an uncommon disease in humans defined by colonization of the luminal surface of colonic epithelial cells with anaerobic spirochetes of the Brachyspiraceace family, which include *Brachyspira aalborgi* (measuring 2-6 µm in length) and *Brachyspira piloscoli* (measuring 4-20 µm in length).^6,7^

The prevalence of IS varies from 2.5% to 32%, depending on geographic locations and diagnostic modalities.^6,8,9^ The reported prevalence of human IS found in rectal biopsy specimens ranges between 2% and 7% in Western countries, whereas the prevalence is considerably higher in patients from India and other parts of Asia.^6^ Of note, the overall prevalence is much lower when the diagnosis is made using stool culture (1.2% to 1.5%^6,10^) compared to that from mucosal biopsies. The highest prevalence of IS was previously reported in homosexual men (30% to 60%) as well as in HIV-positive patients.^3,6,7,11^ However, in a recent study from Japan including 5265 consecutive colorectal biopsies from 4254 patients, the authors found that 5.5% of those with HIV seropositivity had IS compared to 1.7% in those with negative serology.^7^ The lengths of the spirochetes were also significantly longer in HIV-positive patients.^7^

IS is found primarily in the colon, though there have been reported cases in the stomach and small intestine from the early 1900s.^6^ Similar to the case we present, most cases of IS are an incidental finding discovered during a screening/surveillance colonoscopy.^5^ The clinical as well as prognostic significance of IS are debatable. Given the lack of association between the presence of IS and GI symptoms, current theory suggests IS has a commensal relationship with the human host and is part of normal flora.^2,6,12^ However, spirochetes can become pathogenic and invasive in a subset of patients, due to diminishing host defenses or a pathologic factor favoring the virulence of the microorganism.^6^ In symptomatic cases, IS most commonly presents with chronic watery diarrhea and abdominal pain. Most cases are mild. However, some may present with an invasive and rapidly fatal course.^6^

Using PubMed, we searched the English-language literature published between January 1996 and May 2016. The terms utilized in the search were “intestinal spirochetosis” and “human subjects.” Reference lists of the identified articles were also reviewed to find additional cases. The baseline characteristics, clinical presentations, as well as outcomes of these cases are presented in Table 1.

**Table 1.** Clinical Characteristics of Reported Cases With IS From 1996 to 2016^a^.

Year (Reference)	Age (Years)/Sex	Underlying Condition/Risk Factor	Clinical Presentation	Endoscopic Findings	Histologic Findings	Treatment	Outcome
Adult population (18 years of age and older)							
2015^17^	39/male	HIV	Watery, nonbloody diarrhea, abdominal distention	Normal	IS	Penicillin (2 weeks)	Initially, responded well but then developed toxic megacolon 2 years later requiring total colectomy
2015^18^	63/male	Healthy	Asymptomatic, + FOBT	Intestinal stricture of transverse colon	Chronic infective colitis consistent with IS	Metronidazole (2 weeks)	Not effective, pathology showed mucinous adenocarcinoma associated with IS requiring subtotal colectomy
2014^19^	37/male	15-year history of pan-ulcerative total colitis	Diarrhea 2-3 times per day, occasional bloody stools	Mild erosive mucosa in both sigmoid colon and rectum; longitudinal ulcer in transverse colon	IS	Mesalazine + prednisolone	Responsive to mesalazine and prednisolone but difficult to taper prednisolone; improvement after metronidazole
						Metronidazole	
	61/male	20-year history of distal ulcerative colitis	Diarrhea 4-5 times per day, occasional bloody stools	Irregularly shaped ulcer in rectum	IS	Prednisolone	No resolution of ulcer with prednisolone; improvement with metronidazole
						Metronidazole	
2014^20^	60/male	Hepatitis C cirrhosis	Progressive weight loss	Sessile polyp in ascending colon	IS	No treatment	No follow-up information
2011^21^	34/male	Healthy	Abdominal pain, diarrhea	Not performed; CT showed colocolic intussusception	IS; florid lymphoid hyperplasia in submucosa of terminal ileum and ileocecal valve	Right hemicolectomy	Resolution
2010^22^	60/male	Healthy	Lower abdominal pain, loose stools	Mild erythema of cecum and ascending colon	IS	Metronidazole (400 mg ×10 days)	Improvement
2009^23^	Middle-aged	HIV	Soft stools, occasionally bloody	Small polyp in cecum	Tubular adenoma with IS on luminal epithelium	Amoxicillin	No follow-up information
2008^24^	23/male	Healthy	Diarrhea	Patchy edema with areas of erythema and small erosions	Patchy mucosal inflammation and IS	Clarithromycin (800 mg/day ×10 days)	Improvement
2010^25^	68/male	Healthy	Persistent diarrhea	Normal	IS	Metronidazole (750 mg/8 h ×10 days)	Resolution
2007^26^	17 cases in the series/age 4-75	Healthy and those with HIV	Diarrhea, abdominal discomfort, abdominal pain, iron deficiency anemia	All cases with mucosal erosions/hyperemia	Inflammatory cells infiltrate	Metronidazole	Resolution except for one died from pulmonary embolism and one lost to follow-up
2007^27^	31/male	Healthy	Abdominal pain, watery diarrhea	Edematous mucosa with erythematous spots in ascending and transverse colon; sigmoid sessile polyp	IS	Metronidazole (1000 mg/day ×7 days)	Resolution
2006^28^	11 cases in the series/age 29-87	Healthy and those with HIV	Diarrhea and abdominal pain	Normal to extensive area of inflammation	Normal mucosa to inflammatory cells infiltrate and mucosal ulceration	Metronidazole (500 mg PO 4 times per day)	Resolution except 2 with persistent diarrhea, and one subject with abdominal pain but without reported outcome
						Some cases received benzathine penicillin 2.4 million units IM single dose	
2005^29^	62/male	HIV	Flatulence, intestinal hemorrhage	Pan-colonic hypotonic diverticular disease	IS	Penicillin G	Resolution
2004^30^	41/male	HIV, neuropathy, GERD, depression	Abdominal pain, loose stools, hematochezia	Nonspecific inflammation without colitis	IS	Metronidazole	Resolution
2004^31^	57/female	Rectal prolapse	Asymptomatic	Not performed	IS and pneumatosis coli; IS within pneumatic cysts	No information on treatment	No follow-up
2002^32^	78/male	Non-Hodgkin lymphoma	Severe bloody diarrhea, abdominal pain	Not performed	IS	No information on treatment	No follow-up
2001^33^	50/male	Healthy	Diarrhea, abdominal cramping	Normal	IS	Metronidazole	Resolution
2000^34^	57/male	Healthy	Asymptomatic	Two polyps in descending and sigmoid colon	IS	No information on treatment	No follow-up
2000^35^	32/male	Healthy	Bloating, lower abdominal pain, watery diarrhea	Normal	IS	Metronidazole (500 mg 4×/day for 10 days)	Improvement
1998^36^	65/male	Presumed healthy (HIV test not performed)	Weight loss	Red spot on mucosa of cecum, small polyps in descending colon	IS	No treatment	No follow-up
1996^37^	21/female	Healthy; heterosexual	Rectal bleeding	Active proctitis, mild erythema of rectal and colonic mucosa	IS	Hydrocortisone 1% rectal foam	Resolution
	28/male	Healthy; heterosexual	Intermittent nausea and lassitude, weight loss	Patchy erythema in sigmoid colon, intense erythema, mucosal nodularity and friability in distal rectum	IS in rectal biopsy; lymphocytes and plasma cells within lamina propria, no spirochetes on sigmoid biopsy	High fiber diet (unsure etiology of symptoms and thought to have post-infectious IBS)	Improvement
	45/male	Healthy; heterosexual	Colicky pain in left iliac fossa, flatulence, diarrhea	Normal	IS	No treatment (diagnosed with IBS due to uncertain significance of intestinal spirochetosis at that time)	No follow-up
Pediatric population (0-18 years of age)							
2012^38^	13/male	Recurrent aphthous stomatitis	Blood-stained diarrhea, urgency, weight loss	Mucosal edema in sigmoid and rectum	IS	Amoxicillin (2 weeks)	Cessation of rectal bleeding but continuous mucous diarrhea with amoxicillin; resolution with metronidazole
						Metronidazole (10 days)	
2012^39^	14/female	Healthy	Intermittent generalized abdominal pain	Normal	IS	Metronidazole	No follow-up
2010^40^	11/female	HSV, psoriasis, upper airway disease	Intermittent abdominal pain, hematochezia	Normal	IS	Metronidazole (250 mg 3×/day)	No improvement after repeated courses of metronidazole and vancomycin, spirochetes found on repeat endoscopy
						Metronidazole (1000 mg for 2 weeks, 2 months, then 750 mg/day for 2 weeks)	No follow-up information
						Vancomycin (7 days)	
	6/male	Healthy	Stomach cramps, hematochezia, intermittent diarrhea, rectal prolapse, “pencil-thin” stools	Normal	IS	Metronidazole (250 mg 3×/day for 2 weeks)	Mild improvement but continuous alternating constipation with watery diarrhea, continuous regurgitation, rectal prolapse
	11/female	Healthy	Right lower quadrant pain	Not performed	Mild acute appendicitis and IS in resected appendix	Cefoxitin (30 mg/kg/dose × 4 doses)	Resolution
						Appendectomy	
	17/female	Healthy	Relapsing abdominal pain, nausea, vomiting	Performed, no information	Mild eosinophilic inflammatory infiltrate with IS	No treatment	No follow-up
	10/male	Healthy	Periumbilical and epigastric pain, nausea, fever	Not performed	Acute appendicitis and IS in resected appendix	No treatment	No follow-up
2005^41^	9/male	Healthy	Blood mixed in stool, diarrhea	Normal	IS	No therapy	Resolution, spirochetes eradicated
2004^42^	9/male	Healthy	Abdominal pain, diarrhea, hematochezia	Mild erythema of rectal mucosa	IS	Erythromycin (40 mg/kg/day × 10 days)	Resolution
2002^43^	5/female	Enterobiasis	Diarrhea, abdominal pain, occasional blood	Edema in rectum	IS	Erythromycin 40 mg/kg/day × 10 days	Rectal bleeding ceased, recurrent abdominal pain; no follow-up
	7/male	Healthy	Abdominal pain, diarrhea	Slight proctitis	IS	Doxycycline (200 mg for 1 day, then 100 mg/day for 8 days)	Persistent abdominal symptoms, eradication of spirochetes
	4/female	Healthy	Mucus and bloody stools	Proctitis, juvenile polyps	IS	Clarithromycin (50 mg/kg/day × 10 days)	Improvement
	10/female	Healthy	Blood-stained diarrhea	Hyperemic membranes on rectoscopy	IS	Clarithromycin	Resolution
	13/male	Healthy	Abdominal pain, nausea, weight loss, blood-stained stools	Slight inflammation of rectum	IS and HP-positive gastritis	Omeprazole	No improvement
						Clarithromycin, amoxicillin, omeprazole	Improvement with relapse
						Clarithromycin, metronidazole, omeprazole	Sustained improvement
	8/male	Healthy	Abdominal pain	Juvenile polyp	IS	Penicillin V	No improvement
						Erythromycin (40-50 mg/kg/day × 10 days)	Resolution
	15/female	Healthy	Abdominal pain, blood-stained stools	Normal	IS	Clarithromycin 500 mg, BID for 2 weeks	Relieved discomfort,bleeding persisted; spirochetes eradicated
	14/female	Healthy	Abdominal pain	Normal colonoscopy, HP-positive gastritis	IS	Ranitidine + amoxicillin	No improvement
						Metronidazole	No improvement of symptoms, IS eradicated
2001^44^	12/male	Healthy	Vomiting, diarrhea, weight loss	Normal	IS with mild focal colitis	Metronidazole and amoxicillin for 1 week	Resolution
	12/male	Healthy	Abdominal pain	Normal	IS	Penicillin V and metronidazole (1 week)	Symptoms persisted
						Metronidazole (800 mg 3×/day for 1 week)	Improvement
	16/female	Healthy	Right upper quadrant pain	Normal	IS	Metronidazole (10 days)	Resolution
	9.5/female	Healthy	Diarrhea, bright rectal bleeding	Normal	IS	Amoxicillin and metronidazole (10 days)	Resolution

Abbreviations: IS, intestinal spirochetosis; FOBT, fecal occult blood test; CT, computed tomography; PO, per os; IM, intramuscular; GERD, gastroesophageal reflux disease; IBS, irritable bowel syndrome; HSV, herpes simplex virus; BID, twice a day.

a Cases were limited to nonsyphilitic spirochetosis.

One crucial observation in our case is that the presence of colonic spirochetosis is found on mucosa adjacent to colonic polyps. This leads to the question of whether there is any association between IS and colonic polyps. Omori et al conducted a retrospective case-control study to determine the prevalence of IS in sessile serrated adenomas/polyps (SSA/Ps) in 19 SSA/P cases and 172 controls.^13^ They found that the rate of IS was significantly higher in the SSA/P cases (52.6%, 10/19 cases) compared to that in controls (8.1%, 14/172), suggesting the potential association between IS and SSA/Ps. The finding from this study is similar to results from an Italian study in which the authors also proposed an association between IS and hyperplastic/adenomatous colonic polyps.^14^ Further studies are needed to determine the implications of IS and the presence of colonic polyps.

The endoscopic examination of colonic mucosa has limited value in making diagnosis of IS. In a study of 15 cases with biopsy-proven IS, colonoscopic findings were normal in 6 subjects and nonspecific in the remaining cases (7 with polypoid lesions, 1 with erythematous mucosa, and 1 with questionable lesion).^15^ In general, IS cannot be detected with routine colonoscopy. However, a recent study showed the potential of in vivo diagnosis of IS using confocal endomicroscopy with fluorescein sodium as a contrasting agent and Acriflavine hydrochloride,as a topical agent to highlight superficial cell borders and nuclei.^16^ Using this technique, the spirochetes become visible as bright ring-like bands within the lumina of the crypts.^16^ Of note, in clinical practice, IS is normally found coincidentally in biopsies taken from areas of intestinal mucosa with an irregular appearance. However, in the majority of cases, it is discovered during random biopsies of normal appearing colonic mucosa.^5^ The histological apperances of IS on biopsy specimens using H&E stain is a diffuse blue fringe, approximately 3 to 6 µm thick, along the border of the intercryptal epithelial layer (Figures 1 and 2).^5^ The presence of spirochetes can be confirmed with Warthin-Starry stain (Figure 3).

The decision on whether to treat IS should be tailored to the clinical presentation, the severity of the patients’ symptoms, and their immune status.^5,6,11^ IS can either present asymptomatically, as the organisms responsible are thought to have a commensal relationship with normal gut flora, or symptomatically with associated GI symptoms, as the organisms can also have an invasive, pathogenic form (Table 1). For the former presentation and in a patient such as the one we present, a “wait-and-see” observational approach without any interventions is appropriate. For symptomatic patients, medical treatment with metronidazole (500 mg 4 times a day for 10 days) has been shown to be beneficial.^5,6^

In conclusion, IS can be found accidentally from colonic biopsies, and, in most cases, there is no correlation with clinical symptoms. The association of IS and the presence of colonic polyps has been reported, though further investigation is required to confirm these anecdotal findings. Most cases can be followed without specific treatment. For symptomatic cases, metronidazole is an effective treatment of choice.
